# HSP110 Inhibition in Primary Effusion Lymphoma Cells: One Molecule, Many Pro-Survival Targets

**DOI:** 10.3390/cancers15235651

**Published:** 2023-11-29

**Authors:** Roberta Gonnella, Roberta Zarrella, Michele Di Crosta, Rossella Benedetti, Andrea Arena, Roberta Santarelli, Maria Saveria Gilardini Montani, Gabriella D’Orazi, Mara Cirone

**Affiliations:** 1 Department of Experimental Medicine, Sapienza University of Rome, Viale Regina Elena 324, 00161 Rome, Italy; roberta.gonnella@uniroma1.it (R.G.); zarrella.1907329@studenti.uniroma1.it (R.Z.); dicrosta.1844091@studenti.uniroma1.it (M.D.C.); rossella.benedetti@uniroma1.it (R.B.); a.arena@uniroma1.it (A.A.); roberta.santarelli@uniroma1.it (R.S.); mariasaveria.gilardinimontani@uniroma1.it (M.S.G.M.); 2 Department of Neurosciences, Imaging and Clinical Sciences, University G. d’Annunzio, 00131 Chieti, Italy; gdorazi@unich.it

**Keywords:** PEL, HSP110, DDR, apoptosis

## Abstract

**Simple Summary:**

Exploring the impact of heat shock protein (HSP)**** inhibition in cancer may give new insights into the cellular processes that these molecules sustain to promote cancer survival and may accelerate the discovery of more HSP inhibitors to be introduced in clinical trials. In this study, we explored the expression and role of high-molecular-weight HSP110 in the survival of Primary Effusion Lymphoma (PEL) cells. We found that the proper expression of this HSP is required to prevent lysosomal permeabilization, DNA damage, c-Myc downregulation and STAT3 de-phosphorylation. Indeed, HSP silencing strongly reduces PEL cell survival through the dysregulation of these processes that have been found to be interconnected.

**Abstract:**

Heat shock proteins (HSPs) are highly expressed in cancer cells and represent a promising target in anti-cancer therapy. In this study, we investigated for the first time the expression of high-molecular-weight HSP110, belonging to the HSP70 family of proteins, in Primary Effusion Lymphoma (PEL) and explored its role in their survival. This is a rare lymphoma associated with KSHV, for which an effective therapy remains to be discovered. The results obtained from this study suggest that targeting HSP110 could be a very promising strategy against PEL, as its silencing induced lysosomal membrane permeabilization, the cleavage of BID, caspase 8 activation, downregulated c-Myc, and strongly impaired the HR and NHEJ DNA repair pathways, leading to apoptotic cell death. Since chemical inhibitors of this HSP are not commercially available yet, this study encourages a more intense search in this direction in order to discover a new potential treatment that is effective against this and likely other B cell lymphomas that are known to overexpress HSP110.

## 1. Introduction

Heat-shock proteins (HSP) represent a highly conserved family of proteins widely expressed in cancer cells [1]. These cells strongly depend on the expression of HSPs for their survival, given that these molecules are needed for cancer cell adaption to stressful conditions deriving from internal and/or external insults [2]. HSP inhibition might therefore represent a promising strategy to specifically target cancer cells, while sparing normal cells that express a low level of these molecules and less intensely rely on them for cell survival. Among the numerous functions of HSPs, they may stabilize oncogenes, such as c-Myc [3] and mutp53 [4,5], and chaperone kinases involved in the activation of oncogenic pathways such as NFkB [6] and STAT3 [7] or sustain the expression of molecules involved in the DNA damage response (DDR) [8,9], which is essential to preserve genome integrity. Ours and others’ laboratories have shown that STAT3, a molecular pathway activated by IL-6, released by PEL cells, and via the expression of several viral proteins, plays a crucial role in sustaining the survival and proliferation of Primary Effusion Lymphoma (PEL) [10], an aggressive KSHV-associated lymphoma. This lymphoma is also dependent on c-Myc [11,12], an oncogene whose expression regulates, and may be regulated by, STAT3 [13,14]. Regarding DDR, this protective response is particularly important for cancer cells, threatened by their high replication rate and by the exposure to DNA-damaging anti-cancer treatments [15]. Depending on the type of DNA damage which cells undergo, kinases such as ATM, ATR, or DNA-PK, which are sensors of DNA damage, become activated and initiate a signaling cascade that may end up in the execution of DNA repair [16]. This can be mediated by pathways, such as BER, NER, Mismatch repair, or NHEJ or HR, in the case of double-strand DNA breaks [17]. We have recently reported that several HSPs, including HSP27, HSP70, and HSP90, overlap in their capacity to sustain the stability of several DDR molecules in PEL cells, contributing, also for that reason, to the survival of this aggressive B cell lymphoma. Moreover, in the above-mentioned study, we reported that the inhibition of these three HSPs inhibited STAT3 phosphorylation and reduced pro-inflammatory cytokine release, which may not only affect the survival of cancer cells but also contribute to shaping the tumor microenvironment [9]. Among the HSPs, one of the less studied is HSP110, a holdase chaperone, considered a non-canonical HSP70 protein. Compared to the canonical HSP70, it possesses a longer lid segment which may allow more efficient binding to its substrates [18]. Previous studies have shown that HSP110, also called HSP105 or HSPH1, is essential for the survival of Burkitt’s lymphoma (BL), mainly because its inhibition reduces the expression of c-Myc, a transcription factor acknowledged as an HSP110 client and key in driving BL survival [19]. Interestingly, another study reported that knocking down HSP110 results in impairment of the NHEJ DNA repair pathway in colorectal cancer and sensitizes it to genotoxic therapies [20]. The role of HSP110 in PEL cell survival and the possible molecular mechanisms affected by its inhibition have not been explored yet, although these cells have been reported to be dependent for their survival on HSP70, belonging to the same HSP110 family. The involvement of HSP70 in several cancer hallmarks renders cancer cells “addicted” to this HSP, which explains why its targeting is an effective anti-cancer strategy. In the present study, we investigated for the first time the impact of HSP110 targeting in PEL cells. With this aim, we used a silencing approach, given that no chemical inhibitors of this HSP are commercially available. In addition to cell survival, we evaluated the impact of HSP110 knockdown on DNA damage, expression of DDR molecules involved in the HR and NHEJ DNA repair pathways, known to be sustained by HSPs, and c-Myc expression, an oncogene that, among its numerous functions, also plays a role in regulating the expression of some DDR molecules [21]. We also evaluated whether HSP110 silencing could affect lysosome permeabilization and if this effect could be interconnected with the other effects reported above. This investigation was based on a previous study in which we found that HSP70, belonging to the same family as that of HSP110, plays a key role in maintaining lysosome membrane integrity in PEL cells [22].

## 2. Materials and Methods

### 2.1. Cell Cultures and Treatments

BC3 (ATCC, CRL-2277) and BCBL1 (kindly supplied by Prof. P. Monini, National AIDS center, Istituto Superiore di Sanità, Rome, Italy) are human B cell lines derived from Primary Effusion Lymphoma (PEL). They were cultured in RPMI 1640 medium (Sigma-Aldrich, St. Louis, MO, USA, R1780), supplemented with 10% fetal bovine serum (FBS) (Sigma-Aldrich, St. Louis, MO, USA), L-glutamine (2 mM) (Aurogene, Rome, Italy), and streptomycin/penicillin (100 μg/mL) (Aurogene, Rome, Italy) (complete medium) at 37 °C in a 5% CO_2_ humidified incubator. RKO is a human colon cancer cell line that was cultured in DMEM medium (PAN-Biotech, Aidenbach, Germany, cat. n. P04-03600) supplemented with 10% fetal bovine serum (FBS).

BC3 and BCBL1 cells were seeded into 6-well plates at a density of 2 × 10^5^ cells/well with a final volume of 2 mL. Subsequently, the cells were silenced for HSP110 or, in some cases, treated for 24 h with c-Myc Inhibitor (I c-Myc) (50 μM) (Sigma-Aldrich, St. Louis, MO, USA, 475956).

### 2.2. HSP110 Silencing

PEL cells were seeded in six-well plates at a density of 2 × 10^5^/well, in RPMI medium without serum and antibiotics and transfected with 70 pmol of Small Interfering RNA duplex specific for HSP110 (Santa Cruz Biotechnology Inc., Dallas, TX, USA cat. n. sc-35597) and 14 mL of Lullaby reagent (OZBIOSCIENCES Marseille, France, cat. n. LL70500) according to the manufacturer’s instructions. Control siRNA-A was used as a negative control (Santa Cruz Biotechnology Inc., Dallas, TX, USA, cat. n. sc-37007).

In some experiments, PEL cell lines were pre-treated with Pepstatin-A (20 mM) (Santa Cruz Biotechnology Inc., Dallas, TX, USA cat. n. sc-45036) or z-VAD-fmk (100 μM) (Calbiochem, S. Diego, CA, USA cat. n. 219011) for 1 h before transfection with HSP110 siRNA or Adriamycin (0.4 μg/mL) (Adria (EBEWE Pharma, Rome, Italy, S.r.l.).

### 2.3. Trypan Blue Exclusion Assay

After 48 h of treatment, BC3 and BCBL1 cells were stained with trypan blue (Sigma-Aldrich, St. Louis, MO, USA; cat. n. T8154), and live cells, which do not stain, were identified and counted via light microscopy using a Neubauer hemocytometer. The experiments were performed in triplicate and repeated at least three times.

### 2.4. Cell Cycle Analysis

For cell cycle analysis, the DNA content was analyzed using the method of Propidium Iodide (Sigma Aldrich, St Louis. MO, USA; P4170) staining and flow cytometry. Next, 5 × 10^5^ BC3 scramble or siHSP110 with or without z-VAD-fmk (ZVAD) were treated for 48 h, then washed with cold 1 × PBS and fixed in 70% ethanol on ice for at least 1 h. Successively, cells were washed three times with cold 1× PBS and stained with 50 μg/mL PI for 15 min at 37 °C. Then, the DNA content was measured using a BD Biosciences FACSCalibur. Cell debris was excluded from the analysis by increasing the forward scatter threshold. Cells with a DNA content lower than that of G0/G1 cells were considered as apoptotic cells, sub-G1. The data are representative of at least three independent experiments.

### 2.5. Western Blot Analysis

Cells were harvested, washed with phosphate-buffered saline (PBS; Thermofisher Scientific, Waltham, MA, USA, cat. n. 18912014), centrifuged at 1200 rpm (revolutions per minute) for 5 min at room temperature (RT), and finally lysed in RIPA buffer (150 mM NaCl, 1% NP-40, 50 mM Tris-HCl (pH 8), 0.5% deoxycholic acid, 0.1% SDS, protease, and phosphatase inhibitors). The protein concentration was determined by using the Quick Start Bovine Serum Albumin (BSA) assay (Bio-Rad, Hercules, CA, USA; cat n. 5000206). An equal amount of proteins of each sample (10 μg) was subjected to electrophoresis on 4–12% NuPage Bis-Tris gels (Thermo Fisher Scientific, Waltham, MA, USA, cat. n. NP0321BOX) and finally transferred to nitrocellulose membranes (Amersham, Buckinghamshire, UK, cat n. 10600002) for 45 min in tris-glycine buffer. After 1h of blocking in 1× PBS-0.1% Tween20-3% BSA (Applichem, GMBH, Darmstadt, Germany cat n. A1391,0100), the membranes were incubated with specific primary antibodies overnight at 4 °C. The blots were subsequently washed three times with 1× PBS-0.1% Tween 20 and incubated with the suitable HRP-conjugated secondary antibody at a dilution of 1:10,000 for 30 min at room temperature. After three further washes, the membranes were finally subjected to ECL (Advansta, San Jose, CA, USA, cat n. 12045-D20).

### 2.6. Antibodies

The following primary antibodies were used in Western blots: 

Rabbit polyclonal anti-HSP110 (1:200) (Abcam, Cambridge UK, cat. n. ab24503), rabbit polyclonal anti-PARP (1:1000) (Cell Signaling, Danvers, MA, USA, cat n. 9542), rabbit polyclonal anti-phospho STAT3 Tyr705 (1:500) (Cell Signalling Danvers, MA, USA, cat n. 9145), mouse monoclonal anti-STAT3 (1:500) (Santa Cruz Biotechnology Inc., Dallas, TX, USA, cat n. sc-482), mouse monoclonal anti-BRCA-1 (1:1000) (EMD Millipore, Burlington, MA, USA, cat n. OP92), mouse monoclonal anti-pH2AX (Ser 139) (1:500) (Santa Cruz Biotechnology Inc., Dallas, TX, USA, cat n. sc-517348), mouse monoclonal anti-KU70 (1:200) (Santa Cruz Biotechnology Inc., Dallas, TX, USA, cat n. sc-17789), rabbit polyclonal anti-Caspase-8 (1:200) (Proteintech, Manchester UK, cat. n. 13423-1-AP), rabbit polyclonal anti-BID (1:200) (Proteintech, Manchester UK, cat. n. 10988-1-AP), mouse monoclonal anti-p21 (1:500) (Santa Cruz Biotechnology Inc., Dallas, TX, USA, cat n. sc-397), and rabbit polyclonal anti-c-Myc (1:200) (Proteintech, Manchester UK, cat. n. 10828-1-AP).

Mouse monoclonal anti-b-Actin (1:10,000) (Sigma-Aldrich, St. Louis, MO, USA cat n. A5441) was used to detect the loading control.

The goat anti-mouse IgG Peroxidase Conjugate (Sigma-Aldrich, St. Louis, MO, USA cat n. A3673) and the goat anti-rabbit IgG Peroxidase Conjugate (Sigma-Aldrich, St. Louis, MO, USA cat n. DC03L) were used as secondary antibodies. All the primary and secondary antibodies were diluted in 1× PBS-0.1% Tween 20 solution containing 2% BSA.

### 2.7. Acridine Orange Staining

Control PEL cells and cells in which HSP110 was silenced were stained with acridine orange (Thermofisher Scientific Waltham, MA, USA cat. n. A1301) at 100 μg/mL for 30 min at RT, as previously described [22]. Cells were subsequently analyzed using an Apotome Axio Observer Z1 inverted microscope (Zeiss, Oberkochen, Germany), equipped with an AxioCam MRM Rev.3 camera (Zeiss, Oberkochen, Germany) at 40× magnification.

### 2.8. Immunofluorescence

Control and HSP110 silenced BC3 and BCBL1 cells were harvested after 48 h of culture, washed with PBS, and seeded onto multispot microscope slides (Thermofisher Scientific, Waltham, MA, USA, cat. n., 99-910-90), and then air-dried. Cells were fixed with 2% paraformaldehyde (Electron Microscopy Science, Hatfield, PA, USA 157-8) for 20 min, washed 3 times in PBS, permeabilized with 0.2% Triton X-100 (Sigma-Aldrich, St. Louis, MO, USA, cat n. T-8787)–PBS for 5 min at RT, then blocked with 3% bovine serum albumin (BSA)–1% glycine–PBS for 30 min at RT. Samples were incubated with primary antibody (mouse anti-phospo-H2AX Ser-139 dil. 1:500, Santa Cruz Biotechnology Inc., Dallas, TX, USA, cat n. sc-517348 or goat anti-Cathepsin D dil. 1:500, Santa Cruz Biotechnologies Inc., Dallas, TX, USA, cat. n. sc-6487) for 1 h at RT. After washing in PBS, samples were incubated with secondary antibody Cy3-conjugated sheep anti-mouse IgG, (1:1000) (Jackson Immuno Research Labs. West Grove, PA cat n. 515-165-062) or mouse anti-goat IgG TRITC, (1:500) (Santa Cruz Biotechnologies Inc., Dallas, TX, USA, cat. n. sc-2490) in the dark at room temperature for 30 min. Finally, after three washes with PBS, the samples were stained with DAPI (Sigma-Aldrich, St. Louis, MO, USA cat n. D9564). Microscope slides were mounted using PBS-glycerol 1:1 and visualized using a Apotome Axio Observer Z1 inverted microscope (Zeiss, Oberkochen, Germany) equipped with an Axiocam MRM Rev.3 camera at 40× magnification.

### 2.9. Densitometric and Statistical Analysis

Densitometric analysis of Western blots was performed by using the Image J software (1.47 version, NIH, Bethesda, MD, USA), which was downloaded from the NIH website (http://imagej.nih.gov (accessed on 10 February 2022)).

The results are represented by the mean plus standard deviation (SD) of at least three independent experiments, and statistical analyses were performed using Graphpad Prism**^®^** software (Version 9.0.0 (86) Graphpad software Inc., La Jolla, CA, USA). Student’s *t* test or a nonparametric one-way ANOVA test was used to demonstrate statistical significance. Difference was considered as statistically significant when the *p*-value was * < 0.05.

## 3. Results

### 3.1. HSP110 Is Highly Expressed in PEL Cells and Sustains Cell Survival 

**Figure 1:**
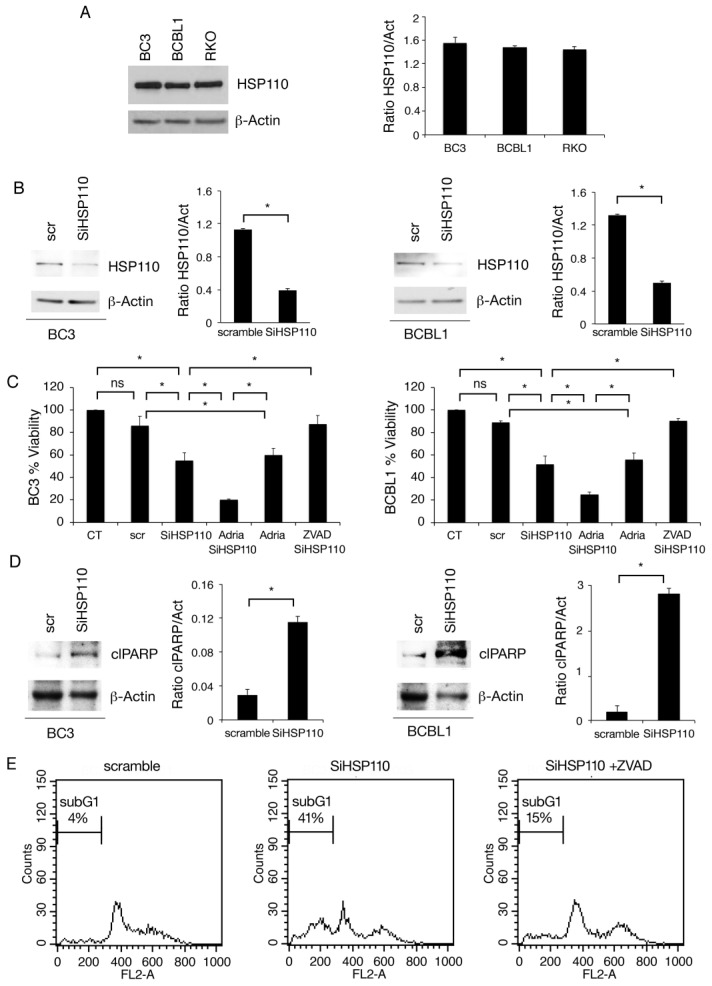
HSP110 is highly expressed in PEL cells and sustains cell survival. (**A**) Protein expression level of HSP110 was evaluated via Western blot analysis in BC3, BCBL1 (PEL), and RKO (colon cancer) cell lines. PEL cells were knocked down for HSP110 or scramble-treated (scr) for 48 h; (**B**) the expression of HSP110 was evaluated via Western blot analysis; (**C**) cell survival was assessed via Trypan blue assay in PEL cells silenced for HSP110 treated with Adriamycin (Adria) or the pan-caspase inhibitor, z-VAD fmk (Z-VAD). The histograms represent the percentage of cell viability relative to the control; data are represented as the mean plus SD of more than three experiments. * *p* value < 0.05, as calculated using an ANOVA test. (**D**) Protein expression level of cleaved PARP (clPARP) was evaluated via Western blot analysis in PEL cells knocked down for HSP110 or scramble-treated (scr). (**A**,**B**,**D**) β-Actin was used as loading control. Histograms represent the mean plus SD of the densitometric analysis of the ratio of HSP110/β-Actin and cleaved-PARP/β-Actin (clPARP/Act). (**E**) Sub-G1 events as evaluated via FACS analysis in BC3 cells scramble-treated or silenced for HSP110, in the presence or in the absence of the pan-caspase inhibitor, z-VAD fmk (Z-VAD). A representative experiment is shown, and the means plus standard deviations of the densitometric analysis of three independent experiments are also reported. * *p* value < 0.05. The uncropped blots are shown in Supplementary Materials.

We first evaluated whether HSP110 was expressed in BC3 and BCBL-1 PEL cells and found that this HSP was highly expressed in both cell lines. A similar expression of HSP110 was found in a colon cancer cell line (RKO) (Figure 1A). As HSP110-specific inhibitors are not commercially available, to investigate the role of HSP110 in PEL cell survival, we silenced it by using specific siRNAs (Figure 1B). As shown in Figure 1C, cell survival was strongly impaired following HSP110 silencing in both PEL cell lines, and its combination with Adriamycin further potentiated the cytotoxic effect of HSP110 silencing. Moreover, pre-treatment with pan-caspase inhibitor z-VAD fmk reduced cell death induced by HSP110 knockdown, suggesting the occurrence of apoptotic cell death (Figure 1C). The type of cell death was then confirmed by the increase in PARP cleavage, induced by HSP110 silencing in PEL cells (Figure 1D), and by the appearance of sub-G1 events in HSP110-silenced PEL cells (Figure 1E). As expected, sub-G1 events were reduced by pre-treating cells with z vad-fmk (Figure 1E).

### 3.2. Lysosomal Permeabilization Contributes to the Cytotoxic Effect of HSP110 Inhibition

**Figure 2:**
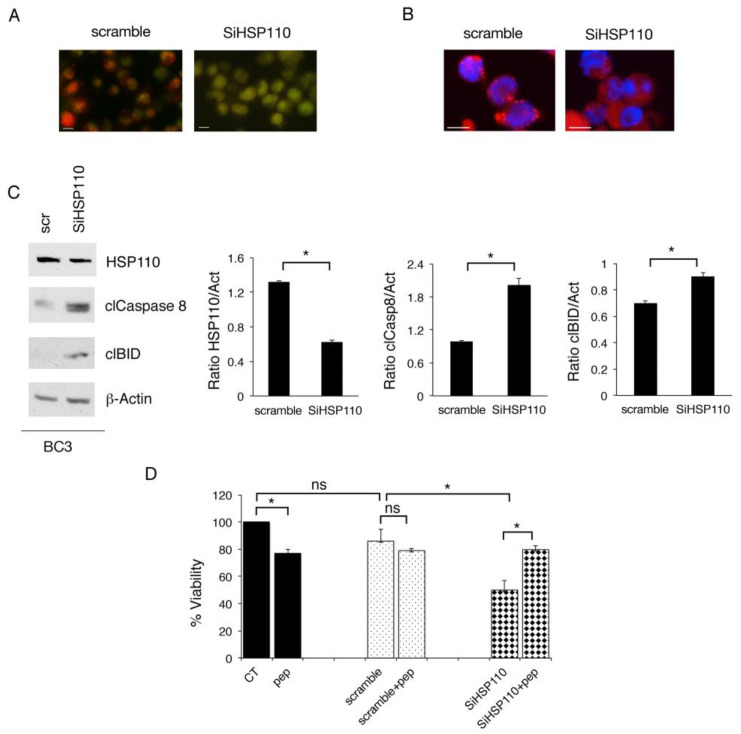
Lysosomal permeabilization contributes to the cytotoxic effect of HSP110 inhibition. (**A**) Lysosomal permeabilization following gene silencing of HSP110 in BC3 cells was assessed via acridine orange (AO) staining. In scramble-treated cells, AO stains lysosomes red since they have intact membranes that maintain an acidic compartment within them. In cells knocked down for HSP110 (SiHSP110), the staining becomes green due to the increase in the pH from lysosomal membrane damage. (**B**) Cellular localization of Cathepsin D in BC3 cells following silencing of HSP110 (SiHSP110) or scramble treatment (scramble) was evaluated in terms of immunofluorescence. (**C**) Western blot analysis of protein expression level of HSP110, cleaved Caspase8 (clCaspase 8), and cleaved BID (clBID) in PEL cells knocked down for HSP110 and scramble-treated (scr) for 48 h. β-Actin was used as loading control. Histograms represent the mean plus SD of the densitometric analysis of the ratio of HSP110/β-Actin, cleaved-Caspase8/β-Actin (clCaspase8/Act), and cleaved BID/β-Actin (clBID/Act). A representative experiment is shown, and the means plus standard deviations of the densitometric analysis of three independent experiments are also reported. * *p* value < 0.05, as calculated via ANOVA test. (**D**) Cell survival of BC3 cells following HSP110 gene silencing, in combination or not with the cathepsin inhibitor, Pepstatin A (pep), assessed using Trypan blue assay. The histograms represent the percentage of cell viability relative to the control; data are represented as the mean plus SD of more than three experiments. * *p* value < 0.05.

The process of lysosomal permeabilization and the release of lysosomal cathepsins into the cytoplasm may contribute to cell death, that may happen either through apoptosis or necroptosis, occurring to the cleavage of BID and caspase 8 activation [17,22]. As we have previously reported that the inhibition of HSP70, belonging to the same family as that of HSP110, induced this effect in PEL cells, here, we evaluated if lysosomal permeabilization could occur in HSP110-silenced cells. With this aim, we used acridine orange (AO), that stains lysosomes red with a normal acid pH while staining the rest of the cytoplasm green. As shown in Figure 2A, red staining was reduced following HSP110 inhibition, signifying a reduction in lysosomal acidity. Accordingly, Cathepsin D, that is localized into the lysosomes, resulting in punctate staining, upon HSP110 silencing gave a diffuse immunofluorescence (Figure 2B). In correlation with the induction of lysosomal permeabilization, the cleavage of BID and caspase 8 increased in these cells (Figure 2C). The contribution of lysosomal cathepsins to cell death following HSP110 knockdown was then confirmed by using the cathepsin inhibitor Pepstatin A that, according to previous findings [22], partially rescued the cell survival of both silenced PEL cell lines (Figure 2D). 

### 3.3. HSP110 Knockdown Induces DNA Damage by Impairing HR and NHEJ DNA Repair, an Effect That Is Also Mediated by the Release of Lysosomal Cathepsins

**Figure 3:**
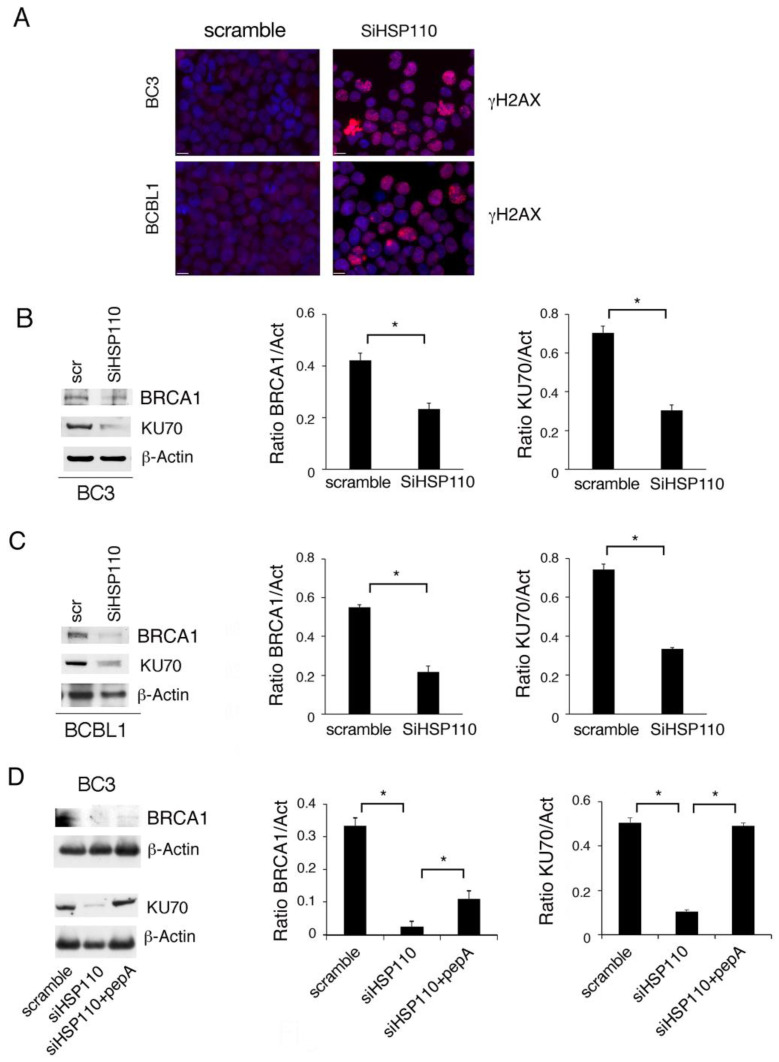
HSP110 knockdown induces DNA damage by impairing DDR molecules. (**A**) The effect of HSP110 gene silencing (SiHSP110) on DNA damage in BC3 and BCBL1 cells was evaluated in terms of immunofluorescence of γH2AX foci. Bars = 10 μm. (**B**,**C**) Western blot analysis of BRCA1 and KU70 protein expression in PEL cells knocked down for HSP110 and scramble-treated (scr) for 48 h. β-Actin was used as loading control. Histograms represent the mean plus SD of the densitometric analysis of the ratio of HSP110/β-Actin (HSP110/Act), BRCA1/β-Actin (BRCA1/Act), and KU70/β-Actin (KU70/Act). (**D**) Protein expression level of BRCA1 and KU70 was evaluated in BC3 cells knocked down for HSP110 untreated or treated with Pepstatin A. β-Actin was used as loading control. Histograms represent the mean plus SD of the densitometric analysis of the ratio of BRCA1/β-Actin (BRCA1/Act) and KU70/β-Actin (KU70/Act). (**B**–**D**) A representative experiment is shown, and the means plus standard deviations of the densitometric analysis of three independent experiments are also reported. * *p* value < 0.05.

Several HSPs play a role in the stability of the molecules involved in DNA repair [8,9]. Here, we explored the impact of HSP110 inhibition on DNA damage and observed that *γ*H2AX-positive foci increased in BC3 and BCBL-1 silenced cells (Figure 3A), suggesting that the reduction in this HSP induced DNA damage. We then correlated this effect with the expression of molecules such as BRCA-1 and Ku70, belonging to HR and NHEJ DNA repair, respectively, and found that the expression level of both was reduced by HSP110 silencing (Figure 3B,C). These results expand the role of HSP110 in sustaining HR DDR, in addition to what has been previously demonstrated in colon cancer cells in which this HSP is involved in NHEJ DNA repair, and thus, its inhibition is able to sensitize these cells to the cytotoxic effects of genotoxic therapies [20]. We then asked if lysosomal cathepsins released in the cytoplasm could contribute to a reduction in Ku70 and BRCA1, as cathepsins have been reported to reduce the stability of DDR molecules such as 53BP1 [23] and also BRCA1 itself [23]. By using the cathepsin inhibitor Pepstatin A, we demonstrated that this was the case, as in HSP110-silenced PEL cells treated by Pepstatin A, the downregulation of Ku70 was counteracted, as well as, partially, that of BRCA-1 (Figure 3D). 

### 3.4. Myc Downregulation via HSP110 Silencing Contributes to wtp53 Activation and DNA Damage Induction in PEL Cells

**Figure 4:**
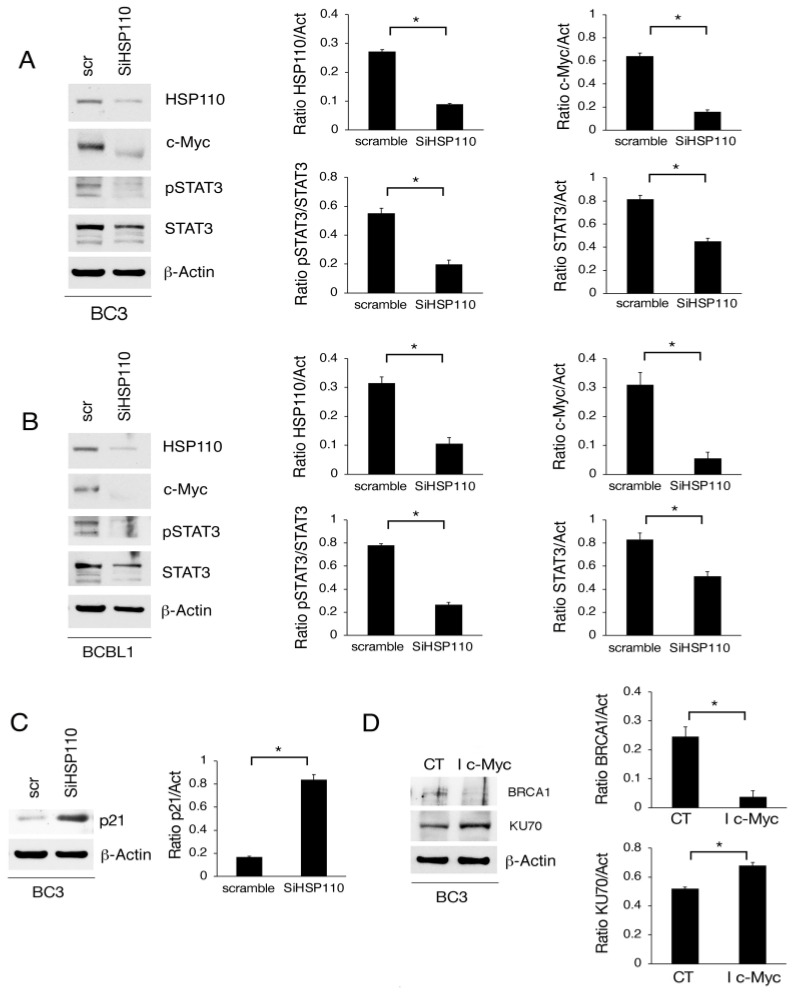
HSP110 silencing induces c-Myc and phospho-STAT3 downregulation, p21 upregulation, and DNA damage induction in PEL cells. (**A**,**B**) Western blot analysis of c-Myc, phospho-STAT3 (Tyr705), and STAT3 protein expression in PEL cells knocked down for HSP110 (SiHSP110) or scramble-treated (scr) for 48 h. (**C**) Western blot analysis of p21 protein expression in BC3 cells following HSP110 gene silencing. (**D**) BRCA1 and KU70 protein expression level was evaluated via Western blot analysis in BC3 cells treated with c-Myc inhibitor (I c-Myc). β-Actin was used as loading control. Histograms represent the mean plus SD of the densitometric analysis of the ratio of HSP110/β-Actin (HSP110/Act), c-Myc/β-Actin (c-Myc/Act), phospho-STAT3/β-Actin (pSTAT3/Act), STAT3/β-Actin (STAT3/Act), p21/β-Actin, BRCA1/β−Actin (BRCA1/Act), and KU70/β-Actin (KU70/Act). A representative experiment is shown, and the means plus standard deviations of the densitometric analysis of three independent experiments are also reported. * *p* value < 0.05.

We then investigated the impact of HSP110 silencing on the c-Myc expression level, as this oncogene plays a key role in driving PEL cell proliferation and it has been reported to be among the numerous HSP110 clients [19]. The results obtained show that c-Myc was downregulated in both PEL cell lines following HSP110 silencing (Figure 4A,B), in agreement with the above-reported study. Several HSPs, including HSP110, have been reported to chaperone STAT3, sustaining its activation [7], which is crucial for PEL cell survival. Accordingly, here, we observed that STAT3 phosphorylation was reduced in HSP110-silenced cells (Figure 4A,B), an effect that could contribute to c-Myc downregulation, with the expression of this oncogene being strictly interconnected with STAT3 activation. 

We have previously shown that c-Myc reduction could lead to p53 activation in BC3, cells that carry endogenous wtp53 [24]. Therefore, here, we explored if the silencing of HSP110 could activate wtp53 in these cells and found that, indeed, the p53 target, p21, was upregulated in HSP110-silenced PEL cells (Figure 4C). This suggests that the activation of an interplay between c-Myc reduction and wtp53 activation, triggered by the downregulation of HSP110, could contribute to inducing PEL cell apoptotic cell death. Moreover, as c-Myc inhibition has been shown to impair DDR [21], here, we evaluated the impact of c-Myc inhibition on BRCA-1 expression and found that this molecule was reduced. Differently, the expression of Ku70, which is involved in the NHEJ pathway, was not affected by c-Myc inhibition (Figure 4D), suggesting that c-Myc downregulation may contribute to the impairment of the HR pathway in HSP110-silenced PEL cells.

## 4. Discussion

One of the few treatments that allow the specific targeting of cancer cells is represented by HSP inhibition [25]. Indeed, these molecules are hyper-expressed in most cancer cells and sustain the activation of several pro-survival pathways such as STAT3 and the expression of oncogenes, such as c-Myc [7,11]. Despite this promising premise, HPS inhibitors, including those able to inhibit HSP90 that stabilizes more than 400 client proteins, involved in key biological processes, have not been introduced yet in clinical trials [26]. Regarding HSP110, whose targeting seems to be particularly efficient at impairing the survival of Burkitt lymphoma [19], there are no commercially available inhibitors either. In this study, we used a silencing approach to investigate the role of HSP110 and showed for the first time that it plays a key role in the survival of PEL cells, an aggressive KSHV-associated lymphoma, characterized by a poor response to conventional chemotherapies [27]. We discovered that, in addition to its already known capacity to reduce c-Myc expression level, HSP110 silencing downregulates key DDR molecules, belonging either to HR and NHEJ, namely BRCA-1 and Ku70. As a consequence, HSP110 inhibition increases DNA damage in PEL cells, as evidenced by the increase in **γ**H2AX-positive foci. Such an effect occurs when the kinases that sense DNA damage become activated [28]. The inhibition of c-Myc can result in DDR dysregulation, reducing the expression of molecules such as BRCA-1 [21]. Accordingly, here, we found that c-Myc inhibition led to a reduction in BRCA-1, suggesting that this mechanism contributes to HR impairment in HSP110-silenced cells. Notably, in this study, we also unveiled for the first time that HSP110 is involved in maintaining lysosomal membrane stability, as demonstrated by AO and cathepsin D staining, similarly to what we and others have previously reported for HSP70 inhibition [22,29]. The role of lysosomal permeabilization in PEL cell death was also confirmed by using Pepstatin A, a cathepsin inhibitor that partially rescued HSP110-silenced PEL cell survival. Interestingly, Pepstatin A counteracted the reduction in Ku70 and partially counteracted that of BRCA-1 induced by HSP110 silencing, thus highlighting a connection between the loss of lysosomal integrity and DNA damage, as a mechanism of cell death induction.

## 5. Conclusions

In conclusion, from this study, it emerges that the inhibition of HSP110 may affect a variety of key processes assuring cell survival, from lysosomal membrane integrity and the HR and NHEJ DNA repair pathways to the expression of c-Myc and the activation of STAT3, aspects that have been found to be somehow interconnected. These findings encourage potentiating the search aimed at developing small molecules able to specifically inhibit HSP110, as a promising strategy to impair the survival of not only PEL cells and other B cell lymphomas, such as BL [19], but possibly also solid cancer cells, such as colon cancer cells in which this HSP has been shown to play an important role in cell survival [30].

## Data Availability

The datasets generated and analyzed during the current study are available from the corresponding author upon reasonable request.

## Supplementary Materials

The following supporting information can be downloaded at: https://www.mdpi.com/article/10.3390/cancers15235651/s1. Full pictures of the Western blots and the densitometry scans are presented in Supplementary Materials file.
