# Supplementary figures and images for: HSP110 Inhibition in Primary Effusion Lymphoma Cells: One Molecule, Many Pro-Survival Targets
Source: Cancers (Basel). 2023 Nov 29;15(23):5651. doi: 10.3390/cancers15235651 (PMC10705194; doi:10.3390/cancers15235651)

FIG1

A

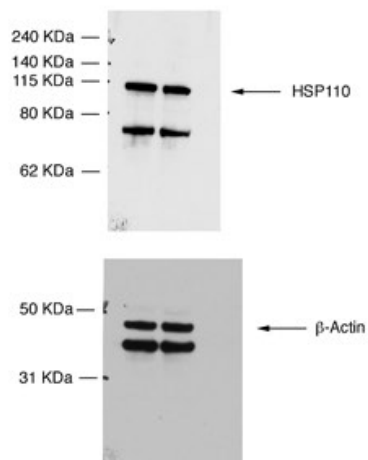

B

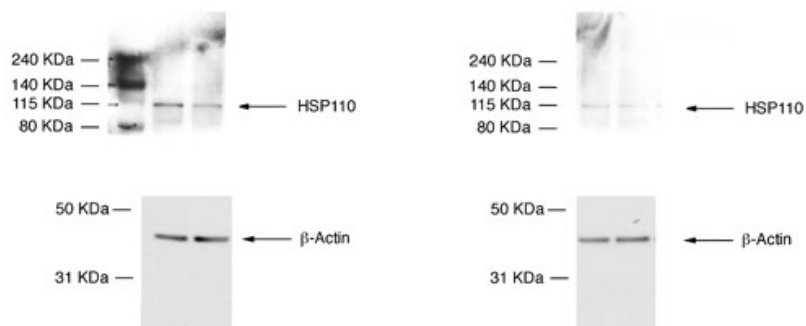

D

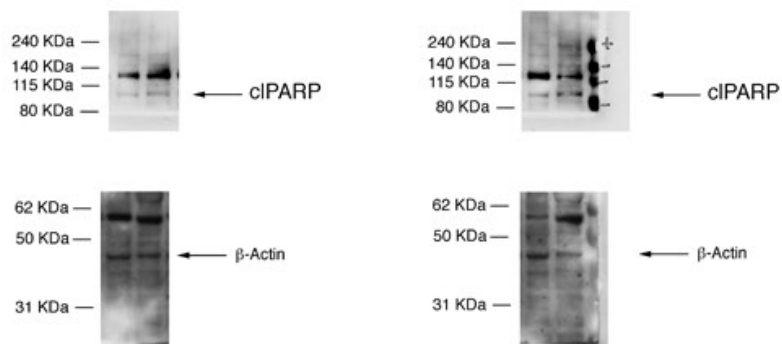

FIG2

C

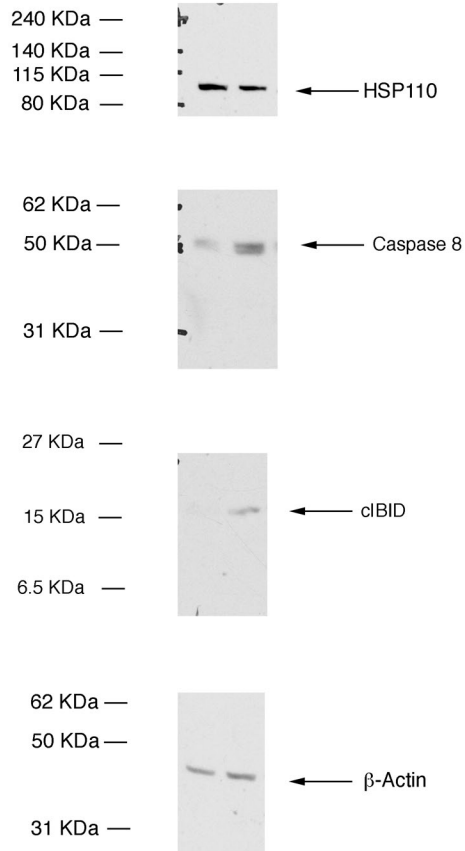

FIG3

B

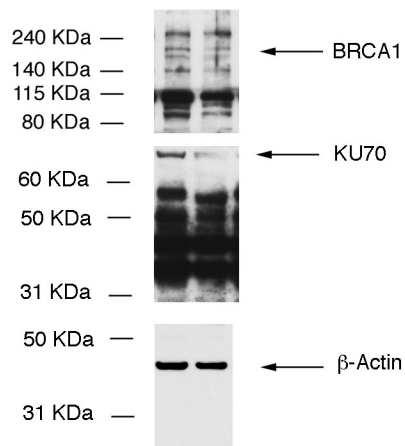

C

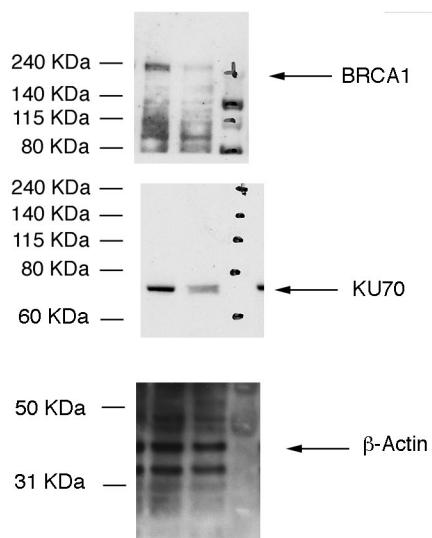

D

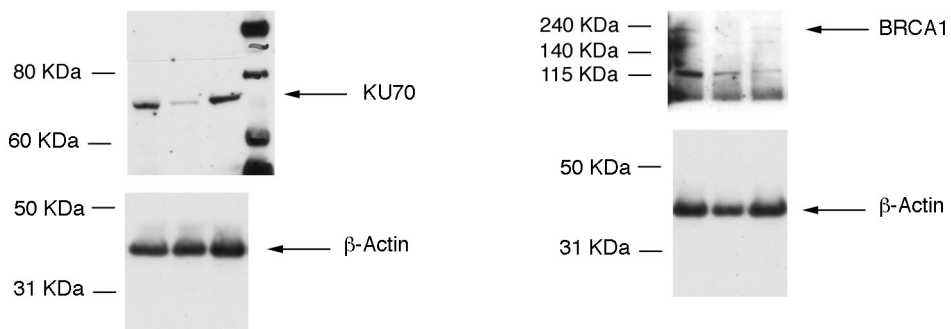

FIG4

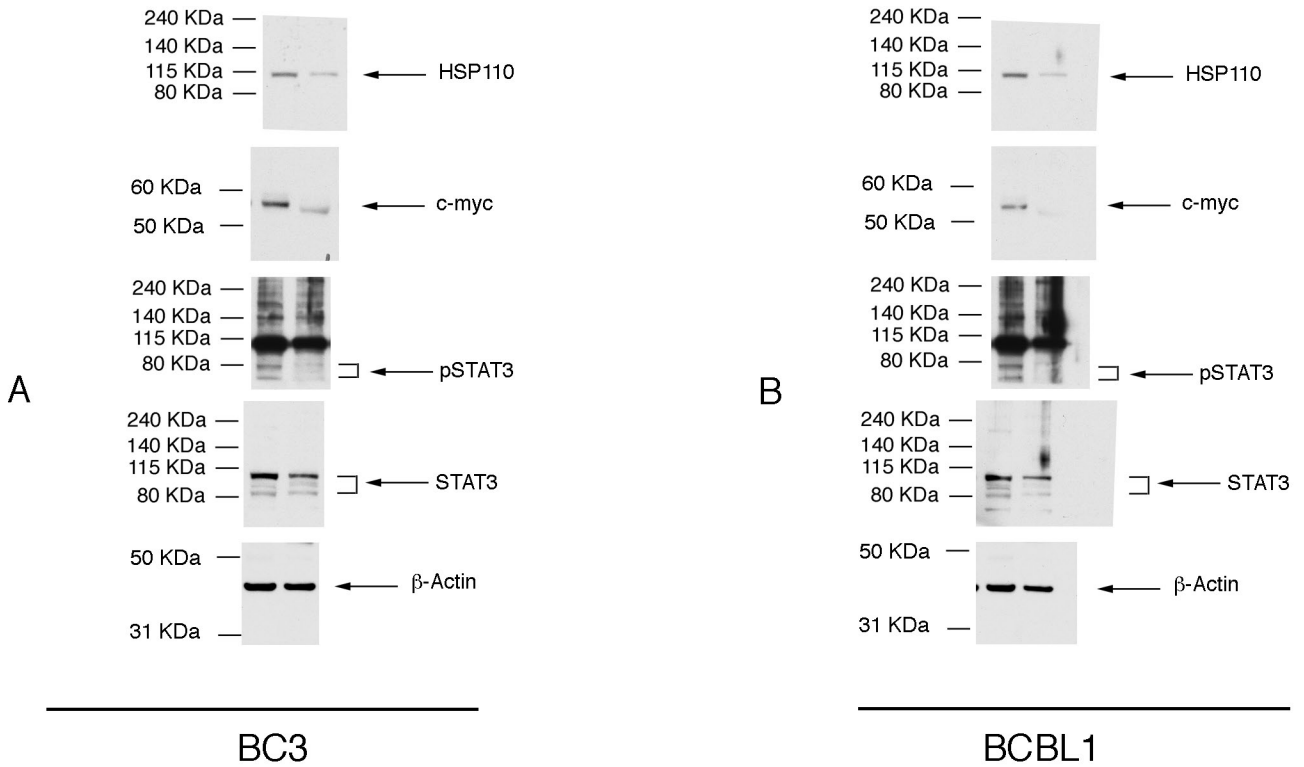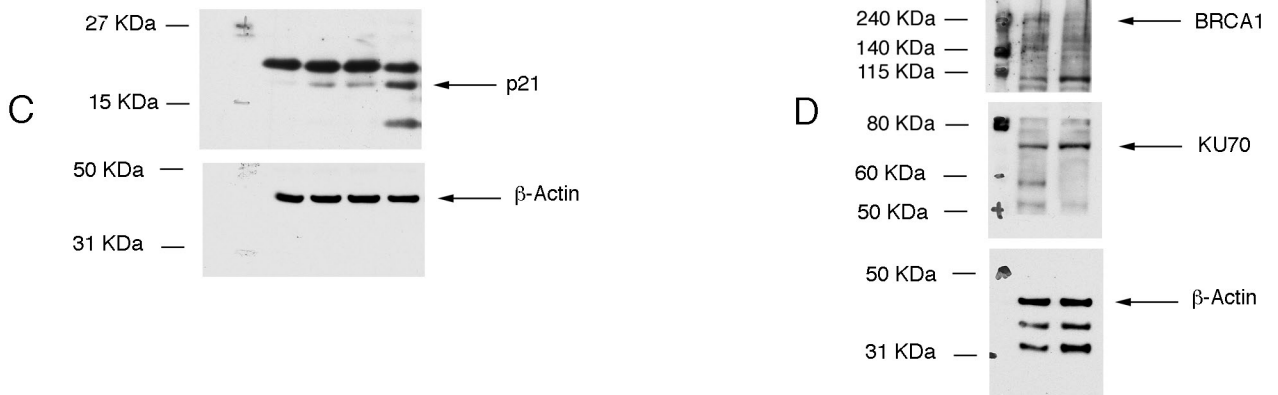

Supplement: Supplementary file 1 [file cancers-15-05651-s001.zip › cancers-2705078-supplementary.pdf]
